# Association between migraine and cognitive impairment

**DOI:** 10.1186/s10194-022-01462-4

**Published:** 2022-07-26

**Authors:** Lihua Gu, Yanjuan Wang, Hao Shu

**Affiliations:** 1 Department of Neurology, Affiliated ZhongDa Hospital, School of Medicine, Southeast University, No. 87 Dingjiaqiao Road, Nanjing, 210009 Jiangsu China; 2 Department of Neurology, Tianjin Huanhu Hospital, Tianjin, China

**Keywords:** Cognition, Migraine, meta-analysis

## Abstract

**Background:**

Previous studies revealed inconsistent results regarding association between migraine and cognitive impairment. In addition, previous studies found inconsistent results regarding the association between migraine and risk of dementia. Thus, the study aimed to make a meta-analysis exploring comparison result in different types of cognitive function between migraine patients and non-migraine subjects. In addition, meta-analysis was made to explore the association between migraine and risk of dementia.

**Methods:**

Articles published before June 2022 were searched in the following databases: PubMed, Web of Science, SCOPUS, EMBASE, EBSCO, PROQUEST, ScienceDirect and Cochrane Database of Systematic Reviews. Results were computed using STATA 12.0 software.

**Results:**

Meta-analysis showed lower general cognitive function and language function in migraine group, compared to no migraine group (general cognitive function: standard mean difference (SMD) = − 0.40, 95% CI = − 0.66 to − 0.15; language: SMD = − 0.14, 95% confidence interval (CI) = − 0.27 to − 0.00), whereas the study showed no significant difference in visuospatial function, attention, executive function and memory between migraine group and no migraine group (visuospatial function: SMD = − 0.23, 95% CI = − 0.53 to 0.08; attention: SMD = − 0.01, 95% CI = − 0.10 to 0.08; executive function: SMD = − 0.05, 95% CI = − 0.16 to 0.05; memory: SMD = − 0.14, 95% CI = − 0.30 to 0.03). In addition, the meta-analysis showed a significant association between migraine and risk of dementia (odds ratio (OR)/relative risk (RR) = 1.30, 95% CI = 1.11 to 1.52).

**Conclusions:**

In conclusion, the meta-analysis demonstrated lower general cognitive function and language function in migraine. In addition, migraine is associated with an increased risk of all-cause dementia, VaD and AD. These results suggest a significant association between migraine and cognitive impairment. Because of the association between migraine and cognitive impairment, neurological physician should be vigilant and effectively intervene in migraineurs with high risk factors of cognitive impairment to prevent the development of cognitive impairment.

**Supplementary Information:**

The online version contains supplementary material available at 10.1186/s10194-022-01462-4.

## Introduction

Migraine has been reported as the sixth most common pathogenesis of disability [1]. In addition, migraine has been reported as the second most common factor associated with disability-adjusted life years worldwide by the Global Burden of Disease study [1]. Migraine is one of the most common pain disorders and its prevalence affects up to 25% of women and 9.4% of men worldwide [2]. In addition, Pompili et al. [3] has systematically documented a strong bidirectional association between migraine and psychiatric disorders. Moreover, the relationship between migraine and psychopathology resulting in enhanced psychosocial impairment has often been clinically discussed rather than systematically studied [4–6]. These studies supported the importance of this emergent research in the field.

Subjective cognitive decline is not unusual in migraine. Although cognitive impairment is identified as the core symptom of migraine, a large amount of migraine patients complain of cognitive impairment, especially deficits in attention and memory. However, previous studies revealed inconsistent results regarding association between migraine and cognitive impairment. Indeed, some studies reported that migraine is associated with a lower cognitive function during both interictal [7] or ictal [8, 9] periods. Wen et al. reported that migraine patients tend to score higher in cognition tests than non-migraine subjects [10]. Conversely, some studies did not show any difference in cognitive function between migraine patients and non-migraine subjects [11–13]. In addition, previous studies found inconsistent results regarding the association between migraine and risk of dementia [14–16]. We hypothesized that migraine patients showed lower general cognitive function, language, visuospatial function, attention, executive function and memory, compared to no migraine group. Additionally, we hypothesized that migraine was significantly associated with risk of dementia. The study aimed to make a meta-analysis exploring comparison result in different types of cognitive function between migraine patients and non-migraine subjects. In addition, meta-analysis was made to explore the association between migraine and risk of dementia.

## Methods

The study was made according to the Preferred Reporting Items for Systematic reviews and Meta-Analysis (PRISMA) guideline [17]. The PRISMA Checklist is included in the Table 1.

**Table 1:** PRISMA 2009 Checklist

Section/topic	#	Checklist item	Reported on page #
**TITLE**			
Title	1	Identify the report as a systematic review, meta-analysis, or both.	1
**ABSTRACT**			
Structured summary	2	Provide a structured summary including, as applicable: background; objectives; data sources; study eligibility criteria, participants, and interventions; study appraisal and synthesis methods; results; limitations; conclusions and implications of key findings; systematic review registration number.	1, 2
**INTRODUCTION**			
Rationale	3	Describe the rationale for the review in the context of what is already known.	2
Objectives	4	Provide an explicit statement of questions being addressed with reference to participants, interventions, comparisons, outcomes, and study design (PICOS).	2, 3
**METHODS**			
Protocol and registration	5	Indicate if a review protocol exists, if and where it can be accessed (e.g., Web address), and, if available, provide registration information including registration number.	3
Eligibility criteria	6	Specify study characteristics (e.g., PICOS, length of follow-up) and report characteristics (e.g., years considered, language, publication status) used as criteria for eligibility, giving rationale.	3
Information sources	7	Describe all information sources (e.g., databases with dates of coverage, contact with study authors to identify additional studies) in the search and date last searched.	3
Search	8	Present full electronic search strategy for at least one database, including any limits used, such that it could be repeated.	3
Study selection	9	State the process for selecting studies (i.e., screening, eligibility, included in systematic review, and, if applicable, included in the meta-analysis).	3
Data collection process	10	Describe method of data extraction from reports (e.g., piloted forms, independently, in duplicate) and any processes for obtaining and confirming data from investigators.	3
Data items	11	List and define all variables for which data were sought (e.g., PICOS, funding sources) and any assumptions and simplifications made.	3
Risk of bias in individual studies	12	Describe methods used for assessing risk of bias of individual studies (including specification of whether this was done at the study or outcome level), and how this information is to be used in any data synthesis.	4
Summary measures	13	State the principal summary measures (e.g., risk ratio, difference in means).	4
Synthesis of results	14	Describe the methods of handling data and combining results of studies, if done, including measures of consistency (e.g., I^2^) for each meta-analysis.	4
Risk of bias across studies	15	Specify any assessment of risk of bias that may affect the cumulative evidence (e.g., publication bias, selective reporting within studies).	4
Additional analyses	16	Describe methods of additional analyses (e.g., sensitivity or subgroup analyses, meta-regression), if done, indicating which were pre-specified.	4
**RESULTS**			
Study selection	17	Give numbers of studies screened, assessed for eligibility, and included in the review, with reasons for exclusions at each stage, ideally with a flow diagram.	4
Study characteristics	18	For each study, present characteristics for which data were extracted (e.g., study size, PICOS, follow-up period) and provide the citations.	4, 5
Risk of bias within studies	19	Present data on risk of bias of each study and, if available, any outcome level assessment (see item 12).	5–7
Results of individual studies	20	For all outcomes considered (benefits or harms), present, for each study: (a) simple summary data for each intervention group (b) effect estimates and confidence intervals, ideally with a forest plot.	5–7
Synthesis of results	21	Present results of each meta-analysis done, including confidence intervals and measures of consistency.	5–7
Risk of bias across studies	22	Present results of any assessment of risk of bias across studies (see Item 15).	5–7
Additional analysis	23	Give results of additional analyses, if done (e.g., sensitivity or subgroup analyses, meta-regression [see Item 16]).	5–7
**DISCUSSION**			
Summary of evidence	24	Summarize the main findings including the strength of evidence for each main outcome; consider their relevance to key groups (e.g., healthcare providers, users, and policy makers).	8
Limitations	25	Discuss limitations at study and outcome level (e.g., risk of bias), and at review-level (e.g., incomplete retrieval of identified research, reporting bias).	9
Conclusions	26	Provide a general interpretation of the results in the context of other evidence, and implications for future research.	9, 10
**FUNDING**			
Funding	27	Describe sources of funding for the systematic review and other support (e.g., supply of data); role of funders for the systematic review.	10

*From:* Moher D, Liberati A, Tetzlaff J, Altman DG, The PRISMA Group (2009). Preferred Reporting Items for Systematic Reviews and Meta-Analyses: The PRISMA Statement. PLoS Med 6(7): e1000097. doi:10.1371/journal.pmed1000097

For more information, visit: **www.prisma-statement.org**

### Search strategy

We searched for articles published before June 2022 in the following databases: PubMed, Web of Science, SCOPUS, EMBASE, EBSCO, PROQUEST, ScienceDirect and Cochrane Database of Systematic Reviews. Included studies explored association between migraine and cognitive impairment. We used the following search terms: (“migraine” OR “hemicrania” OR “cephalagra”) AND (“cognitive impairment” OR “cognitive deficit” OR “dementia” OR “Alzheimer’s disease”).

### Inclusion and exclusion criteria

We included studies investigating the association between migraine and cognitive impairment. We excluded studies according to the following exclusion criteria: 1) Studies which did not provide sufficient information regarding cognitive function in both migraine and healthy controls (HCs); 2) Studies which did not provide sufficient information for odds ratios (ORs) in case-control studies or relative risks (RRs) in cohort studies and their 95% confidence intervals (CIs); 3) Meta-analyses, reviews and case-reports.

### Data extraction

We extracted the following data from finally included studies: Author, publication year, study type, type of migraine, study location, sample size, mean age of patients, gender of patients, disease duration of migraine, attack frequency of migraine, duration of migraine attack, pain intensity, explored cognitive functions, adjusted variables, follow-up time and results.

### Cognitive tests included

According to previous studies [18], neuropsychological examinations were divided into 6 cognitive domains: (1) general cognitive function, (2) language, (3) visuospatial function, (4) attention function, (5) executive function, (6) memory function. General cognitive function was evaluated by the Mini-mental state examination (MMSE) and Montreal cognitive assessment (MoCA). Language function was assessed by Fluency test (phonemic fluency test and verbal fluency test) and Mill hill vocabulary test part A. Visuospatial function was assessed by Rey-Osterrieth complex figure test (ROCFT) and Clock drawing test (CDT). Attention function was evaluated by Trail making test (TMT)-A, Digital Symbol Substitution Test (DSST), Letter digit substitution test (LDST) and Stroop color and word test (SCWT) A, B. Executive function was assessed by Digital span test (DST)-backward, TMT-B, SCWT C and Semantic similarity test. We identified and recorded the mean value and standard deviation (SD) of raw scores of each neuropsychological test. Higher raw scores indicate better cognitive function on almost all the cognitive tests. However, the TMT (A and B) and SCWT (A, B and C) present an exception, as there is a reversed interpretation for the raw scores (where longer time indicates poorer performance). For this reason, the TMT (A and B) and SCWT (A, B and C) scores of the study have been reversed, so that higher scores indicate better performance. The mean value and SD of cognitive scores in migraine and no migraine groups were standardized and reported in relation to the mean value in no migraine groups. Then, each cognitive domain’s standardized score was determined by averaging the standardized scores of relevant tests. Risk of dementia was measured by calculating the incidences of dementia.

### Meta-analysis

We used STATA 12.0 software to compute results. Standardized mean values and SD of cognitive function associated scores were computed. In addition, ORs/RRs and their CIs were computed. We used Q test and I^2^ to evaluate heterogeneities between included studies. We used random effects models to compute results. We used subgroup studies (for different ethnicities and study types) to explore the source of the heterogeneity. We used meta-regression analysis to investigate the source of heterogeneity. We used sensitivity analysis to assess the study stabilization. We used Begg’s test, Egger’s test and funnel plot to assess publication bias.

## Results

### Study characteristics

Supplementary Fig. 1 showed the inclusion and exclusion process. Tables 2 and 3 showed study characteristics. *N* = 22 studies [7, 10–13, 19–35] (including 3295 migraine patients) investigated cognitive function in both migraine and HCs. These studies included *N* = 4 cohort studies and *N* = 18 cross-sectional studies. *N* = 11 studies [14–16, 36–43] included *N* = 3 case-control studies [14, 16, 36] (including 12,871 dementia patients and 56,365 no dementia participants) and *N* = 8 cohort studies [15, 37–43] (including 47,942 migraine patients and 190,024 HCs) investigated the association between migraine and risk of dementia.

**Table 2:** Characteristics of studies regarding comparison in various cognition between migraine group and no migraine group

Study	Study type	Type of migraine	Study location	Sample size	Age (years)	Gender (male%)	Disease duration (years)	Attack frequency (times/year)	Duration of migraine attack (h)	Pain intensity (0–10)	Cognitive function
Zeitlin et al. 1984 [19]	Cross-sectional	migraine	UK	19/19	36.3	NR	NR	NR	NR	NR	Attention, executive function, memory
Jelicic et al. 2000 [12]	Cross-sectional	migraine	The Neth-erlands	99/ 1753	52.0	35%	NR	NR	NR	NR	Attention, memory
Calandre et al. 2002 [20]	Cross-sectional	migraine	Spain	60/30	37.8	36.7%	NR	NR	NR	NR	Executive function, attention, memory
Haverkamp et al. 2002 [21]	Cross-sectional	migraine	Germany	37/17	10	59.4%	NR	NR	NR	NR	Memory
Gaist et al. 2005 [11]	Cohort	MWoA and MwA	Denmark	536/857	56.4 (6.2)	29.8%	NR	NR	NR	NR	Executive function, language, memory, attention
Pearson et al. 2006 [13]	Cross-sectional	MWoA and MwA	UK	74/74	64.4	26%	NR	NR	NR	NR	Executive function, language
Camarda et al. 2007 [7]	Cross-sectional	MwA	Italy	45/90	33.6 (8.6)	31.1%	13.3 (7.7)	34.4 (20.3)	29.6 (20.9)	2.0 (0.8)	General cognitive function, executive function, language, attention
Kalaydjian et al. 2007 [22]	Cross-sectional	Migraine (MwA)	USA	204/1244	47.5 (12.5)	14.7%	NR	NR	NR	NR	General cognitive function
Baars et al. 2010 [23]	Cohort	migraine	the Netherlands	99/ 1724	47.1 (12.9)	36.4%	NR	NR	NR	NR	General cognitive function, executive function, memory
Rist et al. 2011 [24]	Cross-sectional	migraine	France	167/ 938	69.0 (2.9)	15.0%	NR	NR	NR	NR	General cognitive function, attention, executive function, memory
Martins et al. 2012 [25]	Cross-sectional	migraine	Portugal	61/367	61.9	8.19%	NR	NR	NR	NR	memory
Rist et al. 2012 [26]	Cohort	MWoA and MwA	USA	MWoA 248/ MwA 195/ HC 5496	MWoA 65.3 (3.6)/ MwA 65.9 (3.9)	NR	NR	NR	NR	NR	language, executive function
Santangelo et al. 2016 [27]	Cross-sectional	MWoA	Italy	72/72	34.9 ± 11.2	12.5%	15.1 ± 11.6	73.2	NR	NR	General cognitive function, executive function, language, attention, memory
Wang et al. 2016 [28]	Cross-sectional	migraine	China	MV 40/ Simple migraine 40/40	MV 42.7 ± 13.3/ Simple migraine 42.2 ± 15.4	MV 40%/ Simple migraine 45%	NR	NR	NR	NR	General cognitive function, executive function, attention, memory
Wen et al. 2016 [10]	Cross-sectional	migraine	The Netherlands	1021/5399	63.8 (11.1)	18.7%	NR	NR	NR	NR	General cognitive function
Huang et al. 2017 [29]	Cross-sectional	migraine	China	34/24	36.065 ± 10.046	NR	11.25 ± 9.290	42.384	23.4 ± 24.197	6.383 ± 1.670	General cognitive function, executive function, attention, memory, language, visuospatial function
Lo et al. 2017 [30]	Cross-sectional	MWoA and MwA	Italy	14/14/14	MwA 41.28 ± 13.44 MWoA 40.75 ± 11.82	NR	MwA 10.9 ± 3.7 MWoA 12.3 ± 5.8	MwA 60.6 MWoA 72.84	MwA 3.58 ± 2.27 MWoA 4.21 ± 2.99	NR	Attention, language, memory, executive functions
Ferreira et al. 2018 [31]	Cross-sectional	CM	Brazil	30/30	33.7	4.3%	NR	144	NR	8.5	General cognitive function, executive function, attention, memory, language, visuospatial function
Tunç et al. 2018 [32]	Cross-sectional	migraine	Turkey	100/80	36.7 ± 9.4	9%	7.4 ± 7.1	43.2	NR	NR	General cognitive function, attention, memory, language, visuospatial function
Baschi et al. 2019 [33]	Cross-sectional	MWoA	Italy	21/21	29 (4.32)	42.8%	8.57 (3.69)	39.12	NR	NR	Visuospatial memory, Verbal memory, Attention, Executive functions
Karami et al. 2019 [34]	Cross-sectional	migraine	Iran	30/31	25.33	NR	NR	NR	NR	NR	memory
Martins et al. 2020 [35]	Cohort	migraine	Portugal	35/214	61.1 ± 7.4	6%	NR	NR	NR	NR	General cognitive function, executive function, memory

*Abbreviations*: *CM* chronic migraine, *MwA* migraine with aura, *MWoA* migraine without aura, *NR* not reported, *PIQ* performance intelligent quotient, *TIQ* total intelligence quotient, *UK* United Kingdom, *USA* United States, *VIQ* verbal intelligence quotient

**Table 3:** Characteristics of studies regarding association between migraine and risk of dementia

Study	Study type	Type of migraine	Study location	Sample size	Age (years)	Gender (male%)	Adjusted variables	Follow-up time (years)	Result
Tyas et al. 2001 [36]	case-control	migraine	USA	AD 36/HC 658	79.8 ± 5.7	33.3%	age, education and sex, occupational exposure to fumigants/defoliants	NA	AD 3.49 (1.39–8.77)
Chuang et al. 2013 [37]	cohort	migraine	China	Migraine 33,468, no Migraine 133,872	42.4	28.7%	age, sex, diabetes, hypertension, depression, head injury, and CHD	12	All-cause Dementia 1.33 (1.22–1.46)
Pavlovic et al. 2013 [38]	cohort	Migraine	USA	Migraine 136 no Migraine 838	≥70	NA	age, sex, education, depression, hypertension, diabetes, stroke, myocardial infarction, and other heart conditions	NA	All-cause dementia 0.56 (0.27–1.18)
Hagen et al. 2014 [39]	cohort	Migraine (MwA, MWoA)	Norway	Migraine 6740, no headache 29,988	49.7	46.1%	age, sex, education, total HADS score, and smoking	6	VaD 2.9 (1.3–6.6) MwA 3.0 (0.4–22.5) MWoA 2.7 (1.1–6.7)
Tzeng et al. 2017 [40]	cohort	migraine	China	Migraine 1922 no headache 10,860	≥20	31.96	urbanization level, insured premium	10	All-cause Dementia 1.995 (1.572–2.533)
Kostev et al. 2019 [41]	cohort	migraine	Germany	Migraine 3727, no Migraine 3727	67.7	27.1%	age, sex, index year, and co-diagnoses using a propensity score method	10	All-cause dementia 1.43 (1.07–1.78); VaD 1.51 (0.74–2.28); AD 1.87 (1.21–2.52); Unspecified dementia 1.11 (0.57–1.65)
Lee et al. 2019 [14]	case-control	migraine	Seoul	Dementia 11,438 HC 45,752	≥60	32%	age, sex, income, region of residence, hypertension, diabetes, and dyslipidemia	NA	All-cause dementia 1.13 (1.05–1.23)
Morton et al. 2019 [42]	cohort	migraine	Canada	Migraine 200 no Migraine 479	75.9	38.1%	age, sex, education, depression, hypertension, diabetes, stroke, myocardial infarction, and other heart conditions	5	2.97 (1.25–6.61) AD 4.22 (1.59–10.42) VaD 1.52 (0.20–7.23)
George et al. 2020 [15]	cohort	Migraine (MwA, MWoA)	USA	Migraine 1397 no Migraine 9955	60	44.1%	age, race-center, APOE ε4, income and education. BMI, smoking status, hypertension, diabetes, prevalent CHD, drinking status, HDL cholesterol, and total cholesterol	21	All-cause Dementia 1.04 (0.91–1.2) MwA 1.12 (0.88, 1.43) MWoA 1.01 (0.86, 1.19)
Islamoska et al. 2020 [16]	Case-control	Migraine (MwA, MWoA)	Denmark	Migraine 1397 no Migraine 9955	18.3	51%	sex, country of origin, marital status, educational level, headache diagnoses, psychiatric morbidities, and Charlson Comorbidity Index	18.3	All-cause Dementia 1.5 (1.28–1.76) MwA 2.11 (1.48–3.00) MWoA 1.19 (0.84–1.70) All other migraine types 1.48 (1.23–1.78)
Liang et al. 2022 [43]	cohort	Migraine	Sweden	Migraine 352 no Migraine 305	NR	NR	NR	3 or 6	All-cause Dementia 0.49 (0.20–1.21) MWoA 0.66 (0.26–1.66)

*Abbreviations AD*, Alzheimer’s disease, *APOE* apolipoprotein E, *CHD* coronary heart disease, *HADS* Hospital Anxiety and Depression Scale, *HC* healthy control, *MwA* migraine with aura, *MWoA* migraine without aura, *NA* not applicable, *NR* not reported, *USA* United States, *VaD* vascular dementia

### meta-analysis results

#### Comparison in general cognitive function

**Fig. 1:**
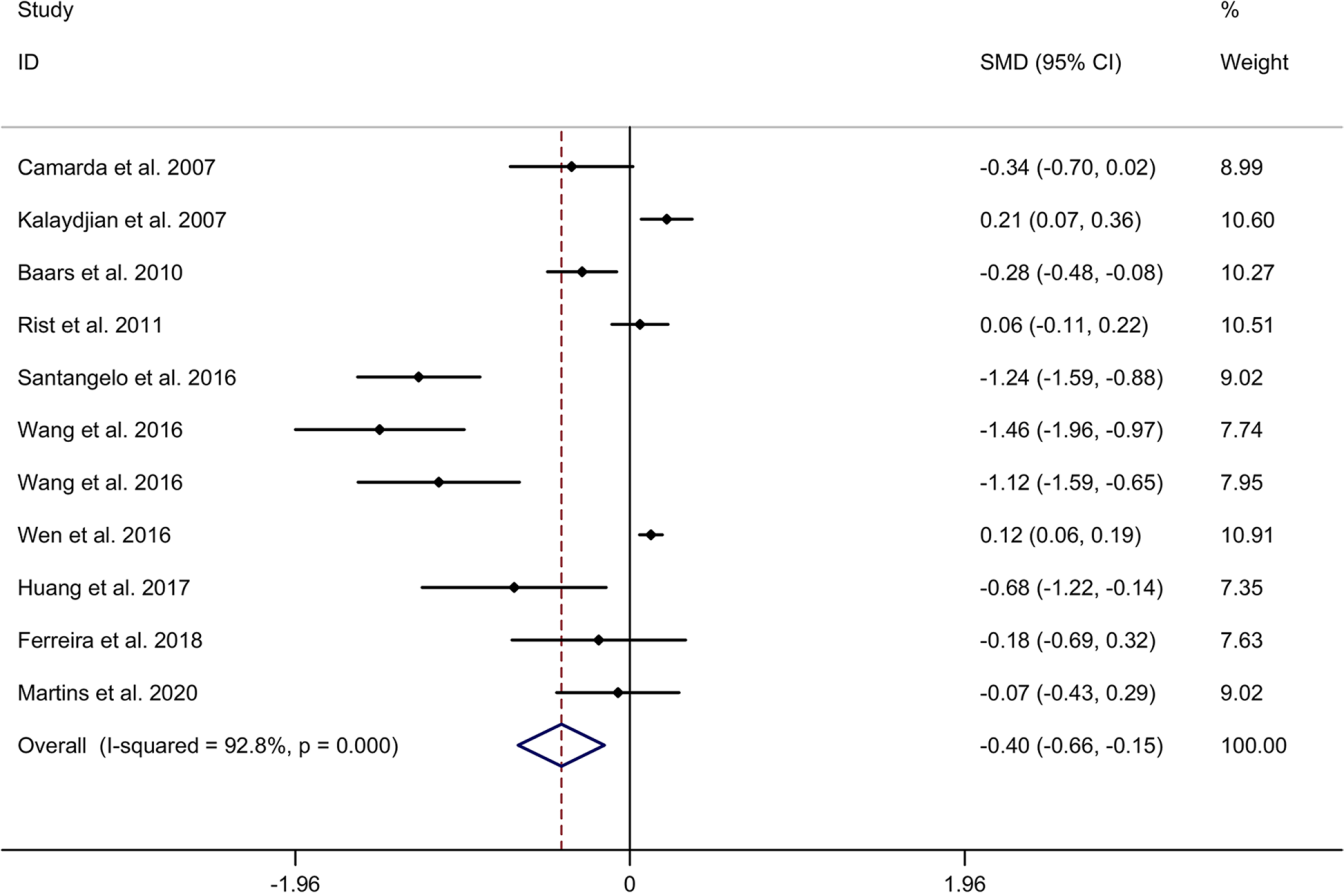
Forest plots regarding comparison in general cognitive function between migraine group and no migraine group. Abbreviations: CI, confidence interval; SMD, standard mean difference

Meta-analysis showed a lower general cognitive function in migraine group, compared to no migraine group with a random effects model (standard mean difference (SMD) = − 0.40, 95% CI = − 0.66 to − 0.15, I^2^ = 92.8%, *p* < 0.001, Fig. 1). Subgroup analysis showed no significant difference in general cognitive function between migraine group and no migraine group in Caucasian, whereas migraine group showed a lower general cognitive function in migraine group, compared to no migraine group in Asian (Supplementary Table 1 and Supplementary Fig. 2. A). Subgroup analysis showed a lower general cognitive function in migraine group, compared to no migraine group in cross-sectional studies (Supplementary Table 2 and Supplementary Fig. 2. B). Meta-regression analysis showed that age of migraine, gender of migraine, disease duration of migraine, attack frequency of migraine and pain intensity were not responsible for the heterogeneity across studies (Supplementary Table 3). Sensitivity analysis indicated no changes in the direction of effect when anyone study was excluded (Supplementary Fig. 3. A). Begg’s test, Egger’s tests and funnel plots indicated a significant risk of publication bias (Supplementary Table 4 and Supplementary Fig. 3. B).

#### Comparison in language function

**Fig. 2:**
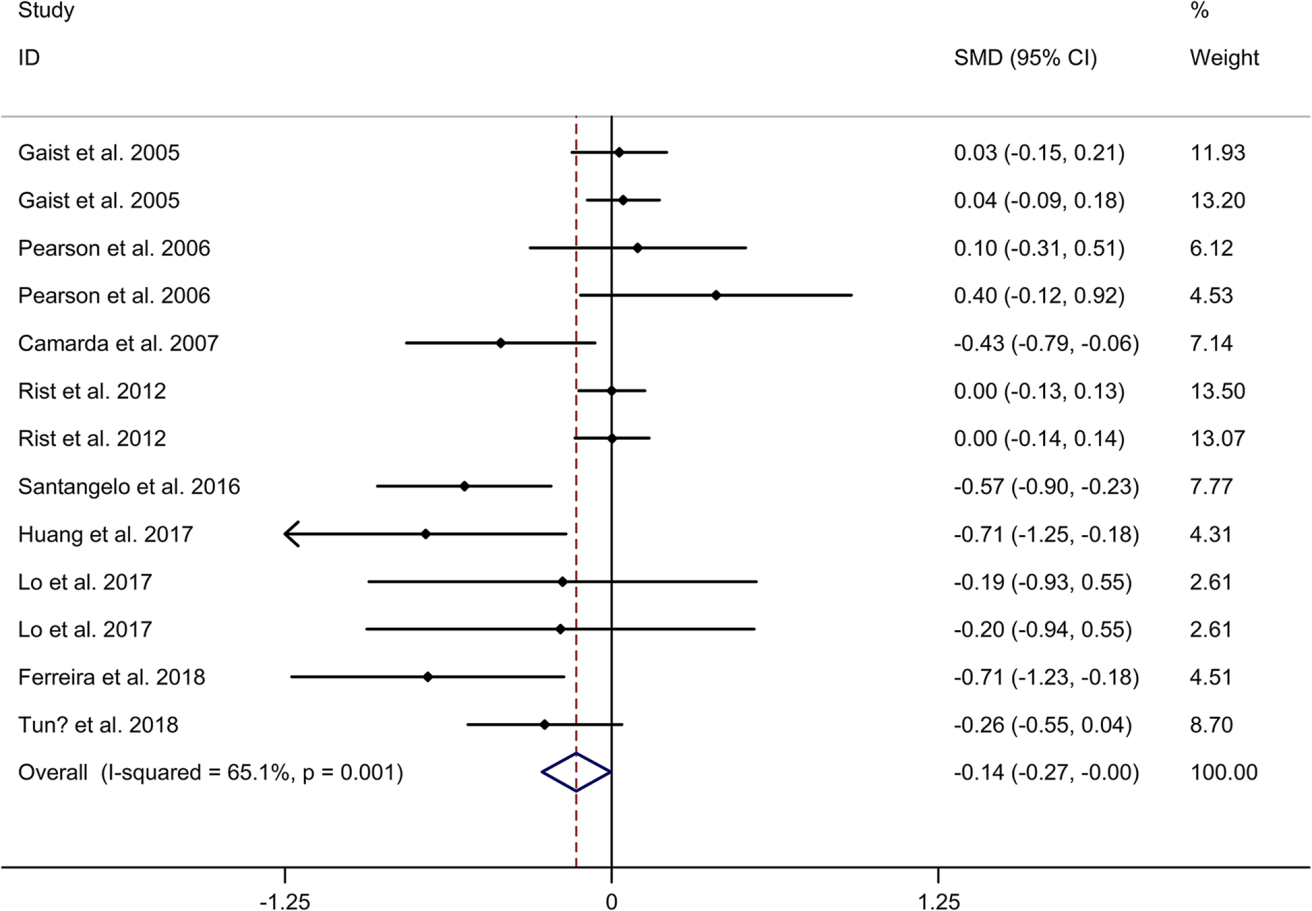
Forest plots regarding comparison in language function between migraine group and no migraine group. Abbreviations: CI, confidence interval; SMD, standard mean difference

Meta-analysis showed a lower language function in migraine group, compared to no migraine group with a random effects model (SMD = − 0.14, 95% CI = − 0.27 to − 0.00, I^2^ = 65.1%, *p* = 0.001, Fig. 2). Subgroup analysis showed no significant difference in language function between migraine group and no migraine group in Caucasian (Supplementary Table 1 and Supplementary Fig. 4. A). Subgroup analysis showed a lower language function in migraine group, compared to no migraine group in cross-sectional studies (Supplementary Table 2 and Supplementary Fig. 4. B). Meta-regression analysis showed that age of migraine was responsible for the heterogeneity across studies, whereas gender of migraine, disease duration of migraine, attack frequency of migraine, duration of migraine attack and pain intensity were not responsible for the heterogeneity across studies (Supplementary Table 3). Sensitivity analysis indicated no changes in the direction of effect when anyone study was excluded (Supplementary Fig. 5. A). Begg’s test, Egger’s tests and funnel plots indicated no significant risk of publication bias (Supplementary Table 4 and Supplementary Fig. 5. B).

#### Comparison in visuospatial function

**Fig. 3:**
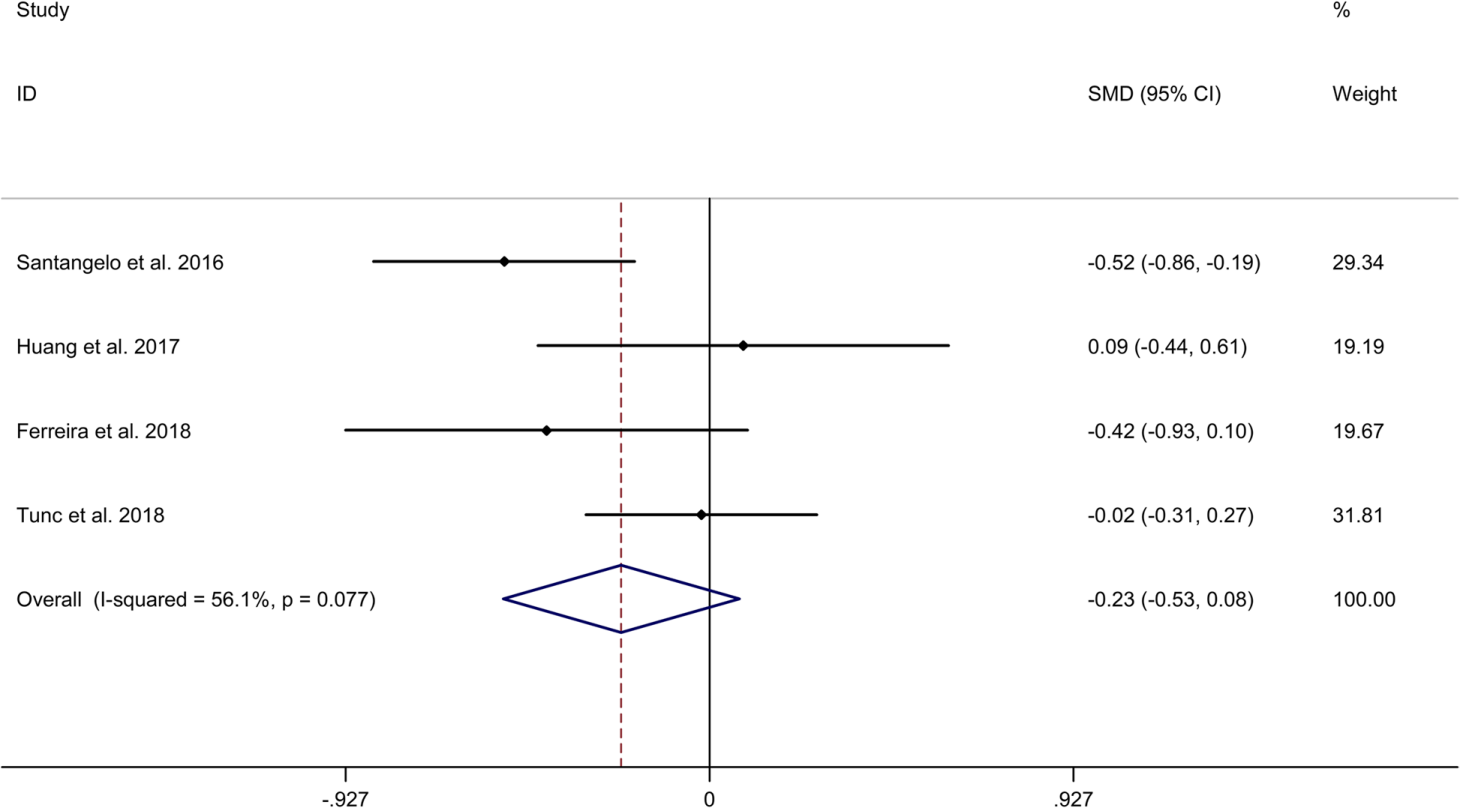
Forest plots regarding comparison in visuospatial function between migraine group and no migraine group. Abbreviations: CI, confidence interval; SMD, standard mean difference

In addition, meta-analysis showed no significant difference in visuospatial function between migraine group and no migraine group with a random effects model (SMD = − 0.23, 95% CI = − 0.53 to 0.08, I^2^ = 56.1%, *p* = 0.077, Fig. 3). Subgroup analysis showed a lower visuospatial function in migraine group, compared to no migraine group in Caucasian (Supplementary Table 1 and Supplementary Fig. 6). Meta-regression analysis showed that age of migraine, gender of migraine, disease duration of migraine and attack frequency of migraine were not responsible for the heterogeneity across studies (Supplementary Table 3).

#### Comparison in attention function

**Fig. 4:**
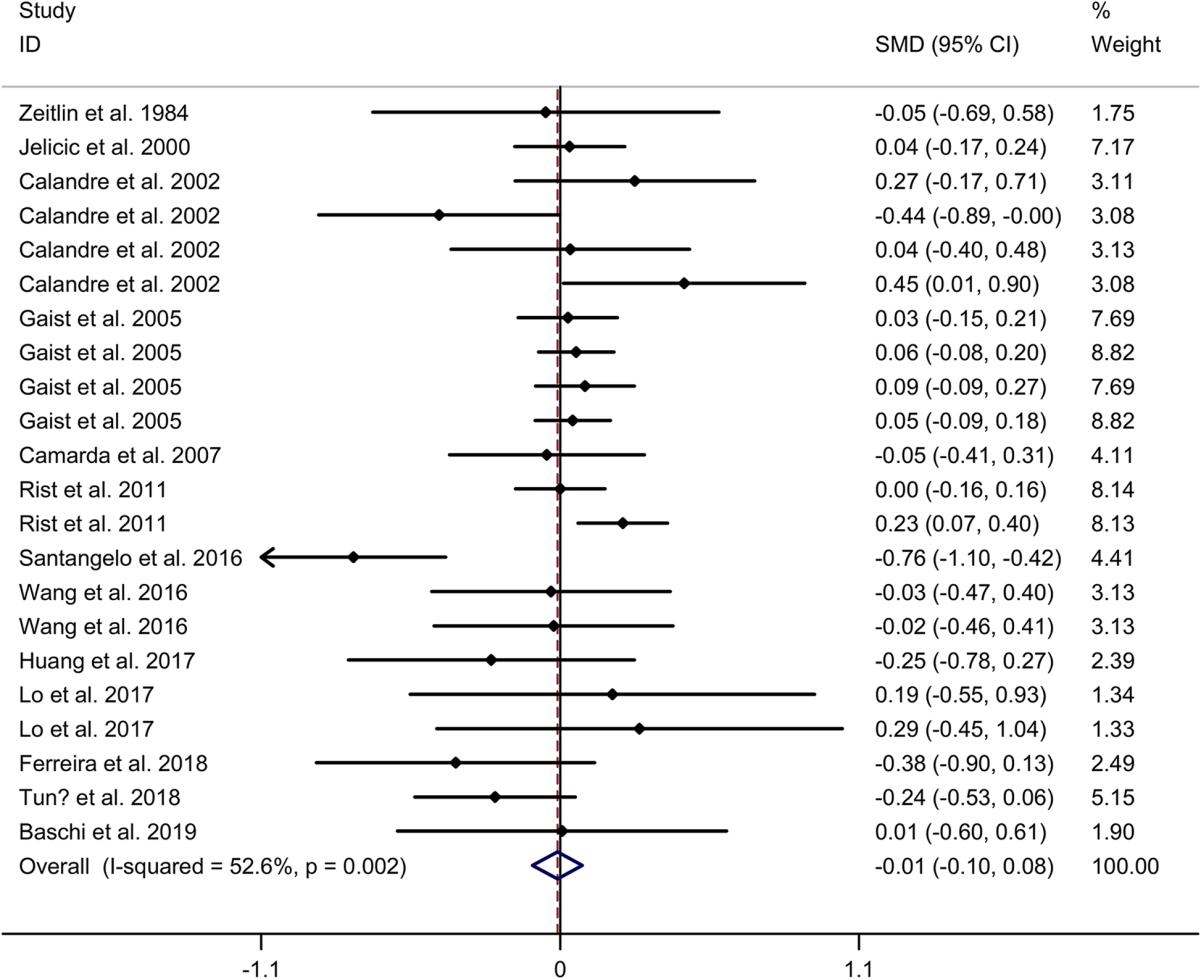
Forest plots regarding comparison in attention between migraine group and no migraine group. Abbreviations: CI, confidence interval; SMD, standard mean difference

However, no significant difference in attention between migraine group and no migraine group with random effects models (SMD = − 0.01, 95% CI = − 0.10 to 0.08, I^2^ = 52.6%, *p* = 0.002, Fig. 4). Subgroup analysis showed no significant difference in attention between migraine group and no migraine group in Caucasian (Supplementary Table 1 and Supplementary Fig. 7. A). Subgroup analysis showed no significant difference in attention between migraine group and no migraine group in cross-sectional studies (Supplementary Table 1 and Supplementary Fig. 7. B). Meta-regression analysis showed that age of migraine and gender of migraine were responsible for the heterogeneity across studies, whereas disease duration of migraine, attack frequency of migraine, duration of migraine attack and pain intensity were not responsible for the heterogeneity across studies (Supplementary Table 3). Sensitivity analysis indicated no changes in the direction of effect when anyone study was excluded (Supplementary Fig. 8. A). Begg’s test, Egger’s tests and funnel plots indicated no significant risk of publication bias (Supplementary Table 4 and Supplementary Fig. 8. B).

#### Comparison in executive function

**Fig. 5:**
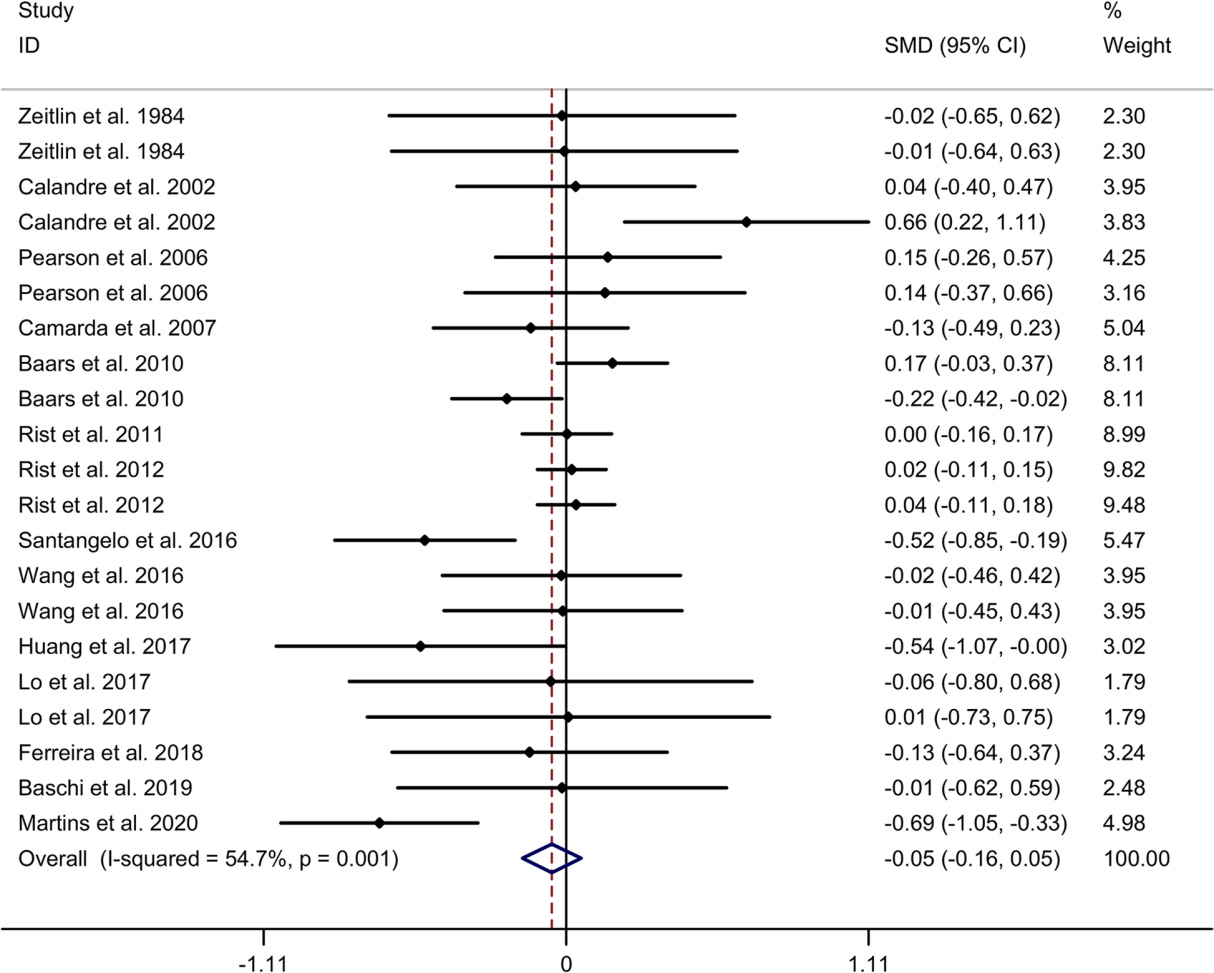
Forest plots regarding comparison in executive function between migraine group and no migraine group. Abbreviations: CI, confidence interval; SMD, standard mean difference

Meta-analysis showed no significant difference in executive function between migraine group and no migraine group with random effects models (SMD = − 0.05, 95% CI = − 0.16 to 0.05, I^2^ = 54.7%, *p* = 0.001, Fig. 5). Subgroup analysis showed no significant difference in executive function between migraine group and no migraine group in Caucasian (Supplementary Table 1 and Supplementary Fig. 9. A). Subgroup analysis showed no significant difference in executive function between migraine group and no migraine group in cross-sectional and cohort studies (Supplementary Table 1 and Supplementary Fig. 9. B). Meta-regression analysis showed that gender of migraine was responsible for the heterogeneity across studies, whereas age of migraine, disease duration of migraine, attack frequency of migraine, duration of migraine attack and pain intensity were not responsible for the heterogeneity across studies (Supplementary Table 3). Sensitivity analysis indicated no changes in the direction of effect when anyone study was excluded (Supplementary Fig. 10. A). Begg’s test, Egger’s tests and funnel plots indicated no significant risk of publication bias (Supplementary Table 4 and Supplementary Fig. 10. B).

#### Comparison in memory function

**Fig. 6:**
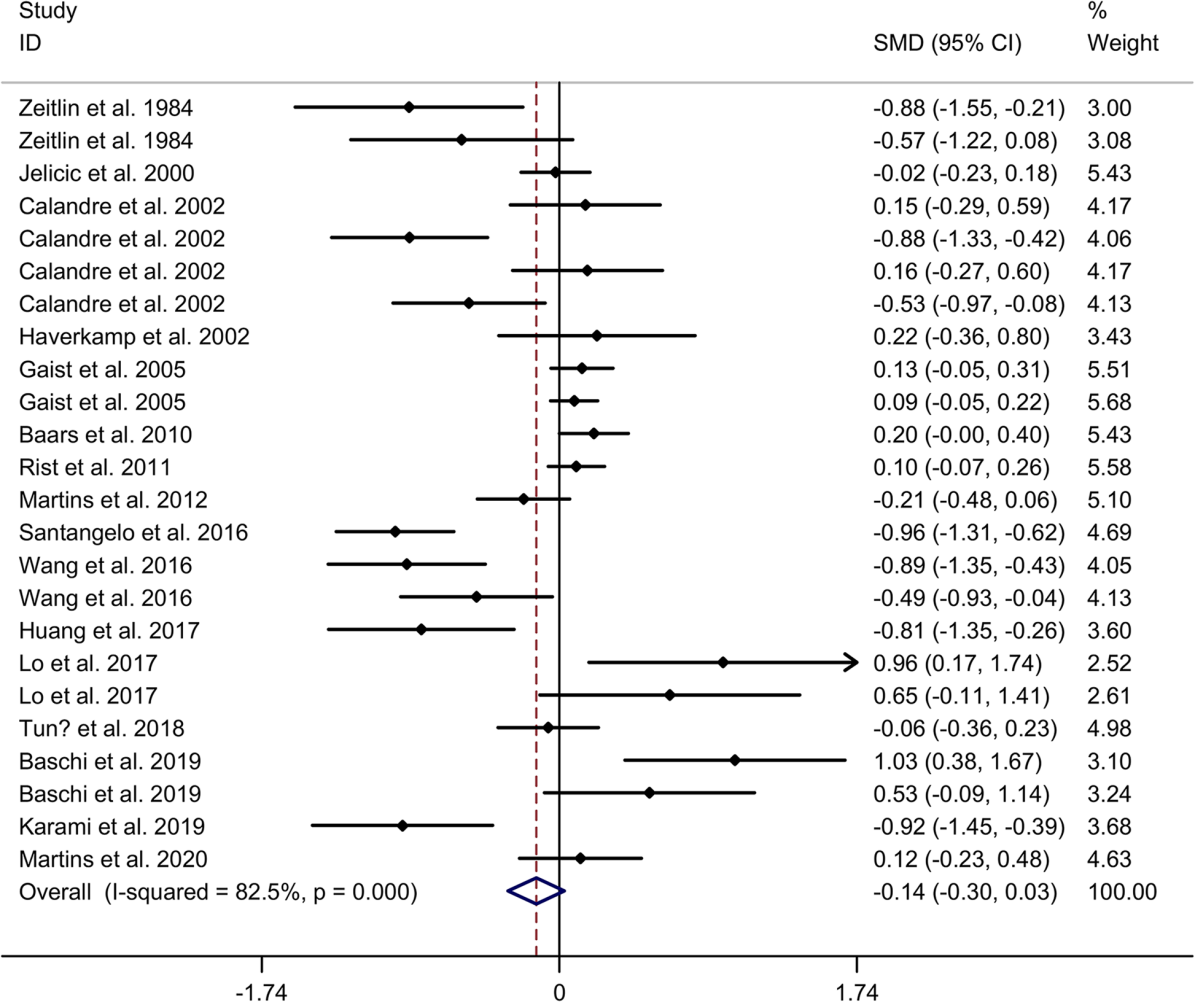
Forest plots regarding comparison in memory between migraine group and no migraine group. Abbreviations: CI, confidence interval; SMD, standard mean difference

Meta-analysis showed no significant difference in memory between migraine group and no migraine group with random effects models (SMD = − 0.14, 95% CI = − 0.30 to 0.03, I^2^ = 82.5%, *p* < 0.001, Fig. 6). Subgroup analysis showed no significant difference in memory between migraine group and no migraine group in Caucasian, whereas migraine group showed a lower memory function, compared to no migraine group in Asian (Supplementary Table 1 and Supplementary Fig. 11. A). Subgroup analysis showed no significant difference in memory between migraine group and no migraine group in cross-sectional studies (Supplementary Table 1 and Supplementary Fig. 11. B). Meta-regression analysis showed that age of migraine, gender of migraine, disease duration of migraine, attack frequency of migraine and duration of migraine were not responsible for the heterogeneity across studies (Supplementary Table 3). Sensitivity analysis indicated no changes in the direction of effect when anyone study was excluded (Supplementary Fig. 12. A). Begg’s test, Egger’s tests and funnel plots indicated no significant risk of publication bias (Supplementary Table 4 and Supplementary Fig. 12. B).

#### Association between migraine and risk of dementia

**Fig. 7:**
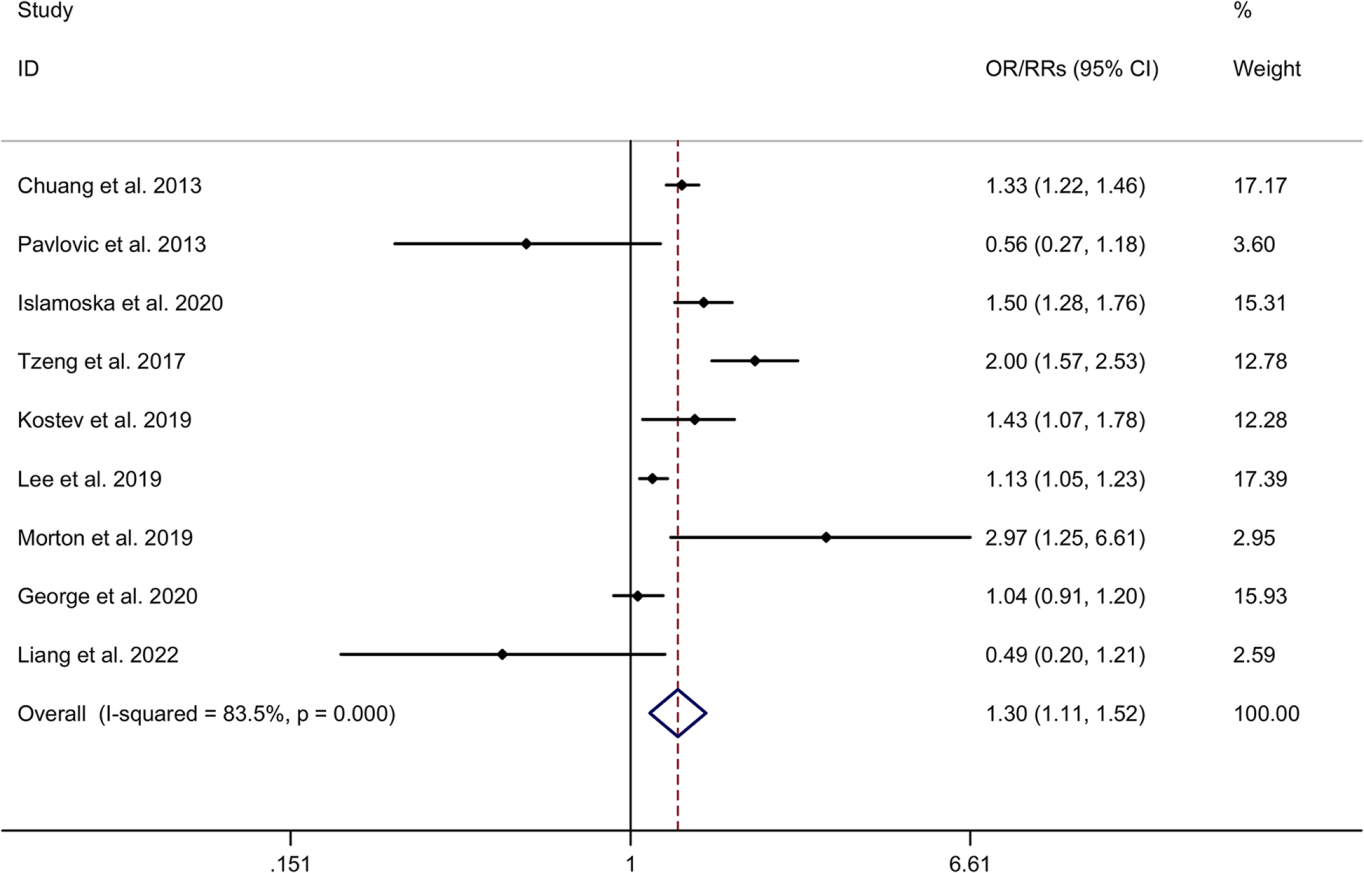
Forest plots regarding association between migraine and risk of dementia. Abbreviations: CI, confidence interval; OR, odds ratio; RR, relative risk

The meta-analysis showed a significant association between migraine and risk of dementia with a random effects model (OR/RR = 1.30, 95% CI = 1.11 to 1.52, I^2^ = 83.5%, *p* < 0.001, Fig. 7). Subgroup analysis showed no significant association between migraine and risk of dementia in Caucasian, whereas a significant association between migraine and risk of dementia was showed in Asian (Supplementary Table 1 and Supplementary Fig. 13. A). Subgroup analysis showed a significant association between migraine and risk of dementia in cohort studies (Supplementary Table 1 and Supplementary Fig. 13. B). Meta-regression analysis showed that age of migraine and gender of migraine were not responsible for the heterogeneity across studies (Supplementary Table 3).

However, the study showed no significant association between migraine without aura (MWoA) and risk of dementia with a random effects model (OR/RR = 1.03, 95% CI 0.89 to 1.19, I^2^ = 0.0%, *p* = 0.453, Supplementary Fig. 14. A). In addition, the study showed significant associations between migraine and risk of vascular dementia (VaD), Alzheimer’s disease (AD) with random effects models (VaD: OR/RR = 1.84, 95% CI 1.18 to 2.88, I^2^ = 0.0%, *p* = 0.423, Supplementary Fig. 14. B; AD: OR/RR = 2.60, 95% CI 1.51 to 4.48, I^2^ = 43.8%, *p* = 0.169, Supplementary Fig. 14. C).

## Discussion

Meta-analysis showed lower general cognitive function and language function in migraine group, compared to no migraine group, whereas the study showed no significant difference in visuospatial function, attention, executive function and memory between migraine group and no migraine group. In addition, the meta-analysis showed a significant association between migraine and risk of dementia.

The present study compared various types of cognition between migraine group and no migraine group. Included studies showed contradictory results on association between migraine and cognitive impairment. Cross-sectional and cohort studies reported worse cognitive function in migraine patients [25] or no association [11–13, 23, 26], whereas some longitudinal studies showed reduced decline of the general cognitive function and executive function in migraine [22, 24]. In addition, Wen et al. [10] reported that migraineurs, particularly migraineurs with aura, tend to score higher in cognition tests than non-migraineurs. These inconsistencies might be caused by different methodological issues including different migraine assessment methods. In addition, clinical features (age, gender, types of migraine, disease duration of migraine, attack frequency of migraine, duration of migraine attack, pain intensity, follow-up duration, headache medication use, diet, sleep, or physical activity, et al.) might be the source of inconsistencies. The effect of age and gender on association between migraine and cognitive impairment has been verified by meta-regression in the present study. More large-scale cohort studies were essential to explore the association between migraine and cognitive impairment.

Up to now, the exact mechanism regarding association between migraine and cognitive impairment is still not fully understood. Recent studies provided information for alterations in brain functional reorganization of cognitive cerebral networks in migraine. These cognitive cerebral networks included default mode network (DMN) [44], executive control network (ECN) [45], visual network [46], et al. The DMN plays an important role in several cognitive processes, such as memory, problem solving and planning [47]. The ECN mainly includes the dorsolateral prefrontal cortex (DLPFC) and the posterior parietal cortex (PPC) [48]. The frontal lobe involves in regulating behavior, complex planning, and learning [49]. Visual processing speed is linked to functional connectivity between right frontoparietal and visual networks [50]. In addition, somatic pain can drive a person to focus on the pain and shift his attention from other cognitive tasks. These mechanisms might contribute to the association between migraine and cognitive impairment.

The meta-analysis showed a significant association between migraine and risk of dementia. The result is corresponding to a recent meta-analysis (including *N* = 9 observational studies) which demonstrated that migraine may be a risk factor for dementia, particularly VaD and AD [51]. Previous studies supported that some vascular risk factors of VaD (including hypertension, diabetes and stroke) could cause migraine [52, 53]. In addition, migraine showed more prevalent in white matter hyperintensities (WMH), which shows an increased risk of dementia both VaD and AD [54, 55], compared to HCs. However, only *N* = 3 studies explored the association between migraine and risk of VaD or AD. Thus, more studies were essential to explore the association between migraine and risk of VaD or AD.

The present meta-analysis showed high heterogeneity between studies investigating association between migraine and risk of dementia. The present study mainly included observational studies, which were both clinically and methodologically inhomogeneous. Thus, high heterogeneity is inevitable and not surprising. Subgroup analysis showed no significant association between migraine and risk of dementia in Caucasian, whereas a significant association between migraine and risk of dementia was showed in Asian. Different ethnicities might be the source of heterogeneity. In addition, other clinical features, such as age, gender, types of migraine, disease duration of migraine, attack frequency of migraine, duration of migraine attack, pain intensity and follow-up duration, might be also the source of heterogeneity. In the present study, we selected studies according to explicit inclusion and exclusion criteria to decrease heterogeneity. However, heterogeneity still exists.

There are some limitations in the study. Firstly, the heterogeneity across studies is unavoidable. The high heterogeneity might have an impact on the reliability of our results. The high heterogeneity might be caused by different methodological issues and clinical features. More large-scale cohort studies were essential to explore the association between migraine and cognitive impairment. Secondly, the study included limited number of studies exploring the association between migraine and risk of VaD or AD. More studies were essential to explore the association between migraine and risk of VaD or AD. Thirdly, some included studies were case-control designed, which might cause recall bias. The recall of migraine may be uncertain and may result in a wrong diagnosis of migraine.

## Conclusion

In conclusion, the meta-analysis demonstrated lower general cognitive function and language function in migraine. In addition, migraine is associated with an increased risk of all-cause dementia, VaD and AD. These results suggest a significant association between migraine and cognitive impairment. Because of the association between migraine and cognitive impairment, neurological physician should be vigilant and effectively intervene in migraineurs with high risk factors of cognitive impairment to prevent the development of cognitive impairment.

## Supplementary Information

**Additional file 1: Figure S1.** Flow of information through the different stages of a meta-analysis.

**Additional file 2: Table S1.** Results of subgroup analysis in different ethnicities.

**Additional file 3: Figure S2.** Subgroup analysis regarding comparison in general cognitive function between migraine group and no migraine group in different ethnicities (A) and study types (B). Abbreviations: CI, confidence interval; SMD, standard mean difference.

**Additional file 4: Table S2.** Results of subgroup analysis in different study types.

**Additional file 5: Table S3.** Results of meta-regression analysis.

**Additional file 6: Figure S3.** Sensitivity analysis (A) and funnel plot (B) regarding comparison in general cognitive function between migraine group and no migraine group.

**Additional file 7: Figure S4.** Subgroup analysis regarding comparison in language between migraine group and no migraine group in different ethnicities (A) and study types (B). Abbreviations: CI, confidence interval; SMD, standard mean difference.

**Additional file 8: Table S4.** Results of publication bias.

**Additional file 9: Figure S5.** Sensitivity analysis (A) and funnel plot (B) regarding comparison in language between migraine group and no migraine group.

**Additional file 10: Figure S6.** Subgroup analysis regarding comparison in visuospatial function between migraine group and no migraine group in different ethnicities. Abbreviations: CI, confidence interval; SMD, standard mean difference.

**Additional file 11: Figure S7.** Subgroup analysis regarding comparison in attention between migraine group and no migraine group in different ethnicities (A) and study types (B). Abbreviations: CI, confidence interval; SMD, standard mean difference.

**Additional file 12: Figure S8.** Sensitivity analysis (A) and funnel plot (B) regarding comparison in attention between migraine group and no migraine group.

**Additional file 13: Figure S9.** Subgroup analysis regarding comparison in executive function between migraine group and no migraine group in different ethnicities (A) and study types (B). Abbreviations: CI, confidence interval; SMD, standard mean difference.

**Additional file 14: Figure S10.** Sensitivity analysis (A) and funnel plot (B) regarding comparison in executive function between migraine group and no migraine group.

**Additional file 15: Figure S11.** Subgroup analysis regarding comparison in memory between migraine group and no migraine group in different ethnicities (A) and study types (B). Abbreviations: CI, confidence interval; SMD, standard mean difference.

**Additional file 16: Figure S12.** Sensitivity analysis (A) and funnel plot (B) regarding comparison in memory between migraine group and no migraine group.

**Additional file 17: Figure S13.** Subgroup analysis regarding association between migraine and risk of dementia in different ethnicities. Abbreviations: CI, confidence interval; OR, odds ratio; RR, relative risk.

**Additional file 18: Figure S14.** Forest plots regarding association between MWoA and risk of dementia, migraine and risk of VaD, migraine and risk of AD. Abbreviations: AD, Alzheimer’s disease; CI, confidence interval; MWoA, migraine without aura; OR, odds ratio; RR, relative risk; VaD, vascular dementia.
