# Supplementary figures and images for: Association between migraine and cognitive impairment
Source: J Headache Pain. 2022 Jul 26;23(1):88. doi: 10.1186/s10194-022-01462-4 (PMC9317452; doi:10.1186/s10194-022-01462-4)

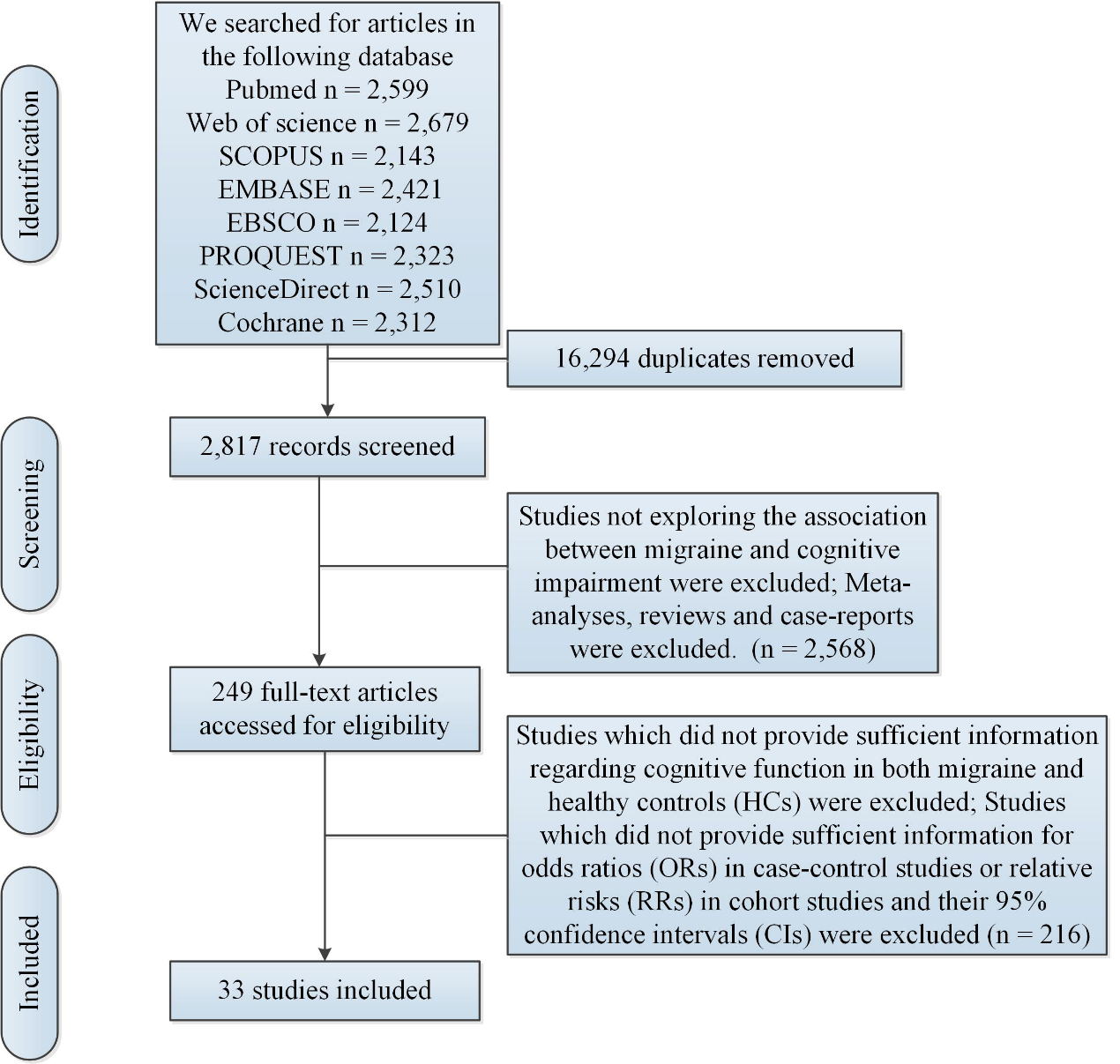


Supplementary figure 1. Flow of information through the different stages of a meta-analysis.

Supplement: Supplementary file 1 — Additional file 1: Figure S1. Flow of information through the different stages of a meta-analysis. [file 10194_2022_1462_MOESM1_ESM.docx]
