# Supplementary material for: Association between migraine and cognitive impairment
Source: J Headache Pain. 2022 Jul 26;23(1):88. doi: 10.1186/s10194-022-01462-4 (PMC9317452; doi:10.1186/s10194-022-01462-4)
Supplement: Supplementary file 2 — Additional file 2: Table S1. Results of subgroup analysis in different ethnicities. [file 10194_2022_1462_MOESM2_ESM.docx]

Supplementary table 1. Results of subgroup analysis in different ethnicities.

| Indicators | Caucasian (SMD or OR/RR, 95%CI) | Asian (SMD or OR/RR, 95%CI) |
| --- | --- | --- |
| comparison in general cognitive function | -0.18 (-0.41, 0.05) | -1.10 (-1.53, -0.67) |
| comparison in language function | -0.10 (-0.23, 0.02) | NA |
| comparison in visuospatial function | -0.27 (-0.47, -0.07) | NA |
| comparison in attention function | -0.00 (-0.10, 0.10) | NA |
| comparison in executive function | -0.04 (-0.16, 0.08) | NA |
| comparison in memory function | -0.02 (-0.18, 0.14) | -0.76 (-1.00, -0.51) |
| association between migraine and risk of dementia | 1.19 (0.90, 1.59) | 1.40 (1.12, 1.74) |

Abbreviations: CI, confidence interval; NA, not applicable; OR, odds ratio; RR, relative risk; SMD, standard mean difference.
