# Supplementary material for: Association between migraine and cognitive impairment
Source: J Headache Pain. 2022 Jul 26;23(1):88. doi: 10.1186/s10194-022-01462-4 (PMC9317452; doi:10.1186/s10194-022-01462-4)
Supplement: Supplementary file 4 — Additional file 4: Table S2. Results of subgroup analysis in different study types. [file 10194_2022_1462_MOESM4_ESM.docx]

Supplementary table 2. Results of subgroup analysis in different study types.

| Indicators | Cross-sectional or case-control studies (SMD or OR/RR, 95%CI) | Cohort studies (SMD or OR/RR, 95%CI) |
| --- | --- | --- |
| comparison in general cognitive function | -0.46 (-0.76, -0.16) | NA |
| comparison in language function | -0.30 (-0.53, -0.07) | NA |
| comparison in attention function | -0.05 (-0.19, 0.09) | NA |
| comparison in executive function | -0.03 (-0.17, 0.10) | -0.09 (-0.28, 0.10) |
| comparison in memory function | -0.20 (-0.42, 0.02) | NA |
| association between migraine and risk of dementia | NA | 1.29 (1.02, 1.64) |

Abbreviations: CI, confidence interval; NA, not applicable; OR, odds ratio; RR, relative risk; SMD, standard mean difference.
