# Supplementary material for: Association between migraine and cognitive impairment
Source: J Headache Pain. 2022 Jul 26;23(1):88. doi: 10.1186/s10194-022-01462-4 (PMC9317452; doi:10.1186/s10194-022-01462-4)
Supplement: Supplementary file 5 — Additional file 5: Table S3. Results of meta-regression analysis. [file 10194_2022_1462_MOESM5_ESM.docx]

Supplementary table 3. Results of meta-regression analysis.

| Indicators | age of migraine | gender of migraine | disease duration of migraine | attack frequency of migraine | duration of migraine attack | pain intensity |
| --- | --- | --- | --- | --- | --- | --- |
| comparison in general cognitive function | 0.155 | 0.187 | 0.590 | 0.722 | NA | 0.946 |
| comparison in language function | 0.023 | 0.876 | 0.264 | 0.304 | 0.502 | 0.492 |
| comparison in visuospatial function | 0.171 | 0.873 | 0.377 | 0.348 | NA | NA |
| comparison in attention function | 0.005 | 0.029 | 0.402 | 0.432 | 0.436 | 0.471 |
| comparison in executive function | 0.407 | 0.037 | 0.291 | 0.966 | 0.678 | 0.815 |
| comparison in memory function | 0.670 | 0.469 | 0.243 | 0.696 | 0.153 | NA |
| association between migraine and risk of dementia | 0.823 | 0.947 | NA | NA | NA | NA |
| association between MWoA and risk of dementia | NA | NA | NA | NA | NA | NA |
| association between migraine and risk of VaD | 0.423 | 0.429 | NA | NA | NA | NA |
| association between migraine and risk of AD | 0.329 | 0.314 | NA | NA | NA | NA |

Abbreviations: AD, Alzheimer’s disease; MWoA, migraine without aura; NA, not applicable; PIQ, performance intelligent quotient; TIQ, total intelligence quotient; VaD, vascular dementia; VIQ, verbal intelligence quotient.
