# Supplementary material for: Association between migraine and cognitive impairment
Source: J Headache Pain. 2022 Jul 26;23(1):88. doi: 10.1186/s10194-022-01462-4 (PMC9317452; doi:10.1186/s10194-022-01462-4)
Supplement: Supplementary file 7 — Additional file 7: Figure S4. Subgroup analysis regarding comparison in language between migraine group and no migraine group in different ethnicities (A) and study types (B). Abbreviations: CI, confidence interval; SMD, standard mean difference. [file 10194_2022_1462_MOESM7_ESM.docx]

Supplementary table 4. Results of publication bias.

| Indicators | *p* value of Begg’s test | *p* value of Egger’s test |
| --- | --- | --- |
| comparison in general cognitive function | 0.062 | 0.004 |
| comparison in language function | 0.180 | 0.052 |
| comparison in attention function | 0.822 | 0.223 |
| comparison in executive function | 0.450 | 0.498 |
| comparison in memory function | 0.637 | 0.140 |

Abbreviations: AD, Alzheimer’s disease; MWoA, migraine without aura; NA, not applicable; PIQ, performance intelligent quotient; TIQ, total intelligence quotient; VaD, vascular dementia; VIQ, verbal intelligence quotient.
