# Supplementary material for: Association between migraine and cognitive impairment
Source: J Headache Pain. 2022 Jul 26;23(1):88. doi: 10.1186/s10194-022-01462-4 (PMC9317452; doi:10.1186/s10194-022-01462-4)
Supplement: Supplementary file 11 — Additional file 11: Figure S7. Subgroup analysis regarding comparison in attention between migraine group and no migraine group in different ethnicities (A) and study types (B). Abbreviations: CI, confidence interval; SMD, standard mean difference. [file 10194_2022_1462_MOESM11_ESM.docx]

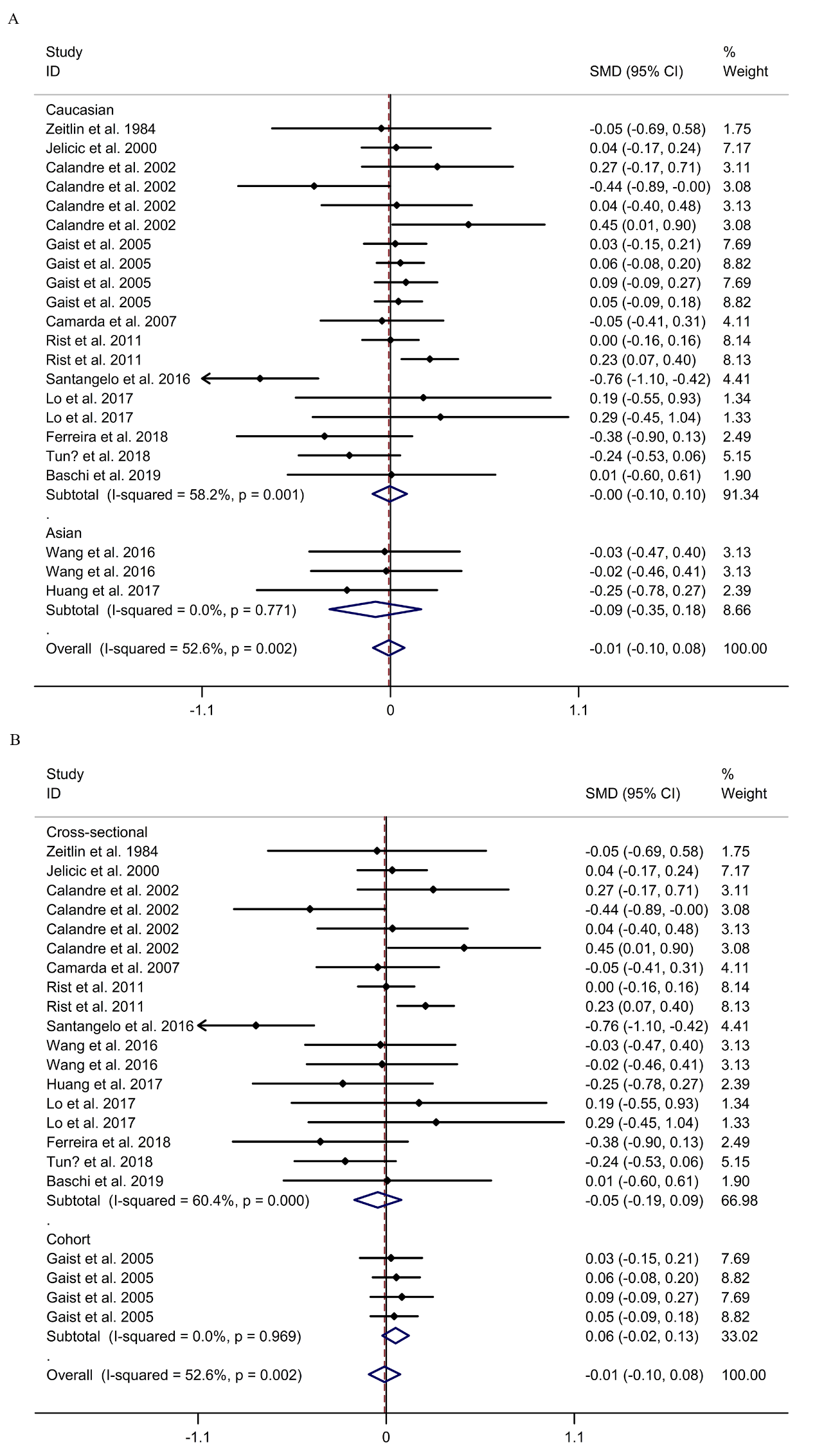


Supplementary figure 7. Subgroup analysis regarding comparison in attention between migraine group and no migraine group in different ethnicities (A) and study types (B). Abbreviations: CI, confidence interval; SMD, standard mean difference.
