# Supplementary material for: Association between migraine and cognitive impairment
Source: J Headache Pain. 2022 Jul 26;23(1):88. doi: 10.1186/s10194-022-01462-4 (PMC9317452; doi:10.1186/s10194-022-01462-4)
Supplement: Supplementary file 12 — Additional file 12: Figure S8. Sensitivity analysis (A) and funnel plot (B) regarding comparison in attention between migraine group and no migraine group. [file 10194_2022_1462_MOESM12_ESM.docx]

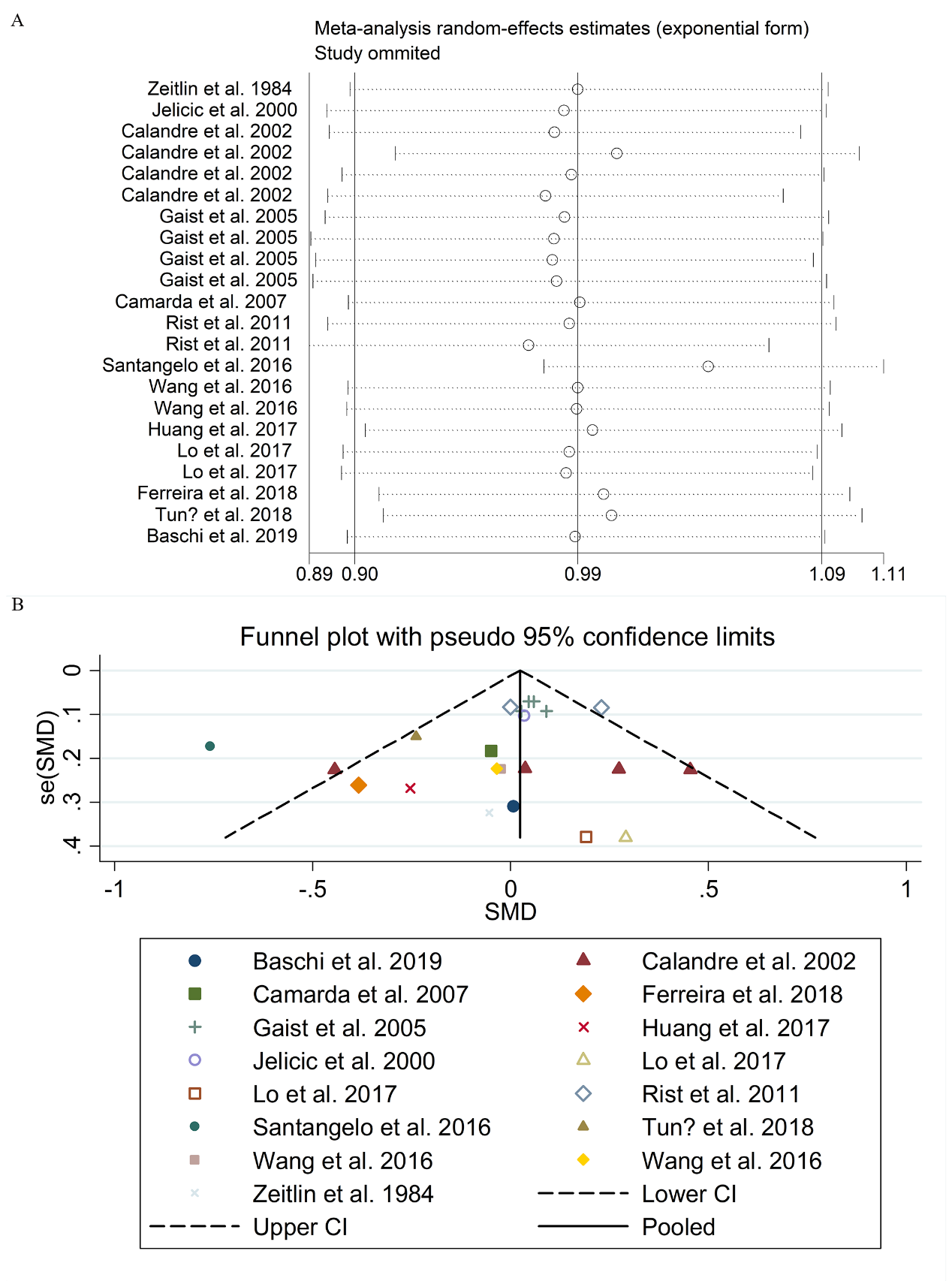


Supplementary figure 8. Sensitivity analysis (A) and funnel plot (B) regarding comparison in attention between migraine group and no migraine group.
