# Supplementary material for: Association between migraine and cognitive impairment
Source: J Headache Pain. 2022 Jul 26;23(1):88. doi: 10.1186/s10194-022-01462-4 (PMC9317452; doi:10.1186/s10194-022-01462-4)
Supplement: Supplementary file 17 — Additional file 17: Figure S13. Subgroup analysis regarding association between migraine and risk of dementia in different ethnicities. Abbreviations: CI, confidence interval; OR, odds ratio; RR, relative risk. [file 10194_2022_1462_MOESM17_ESM.docx]

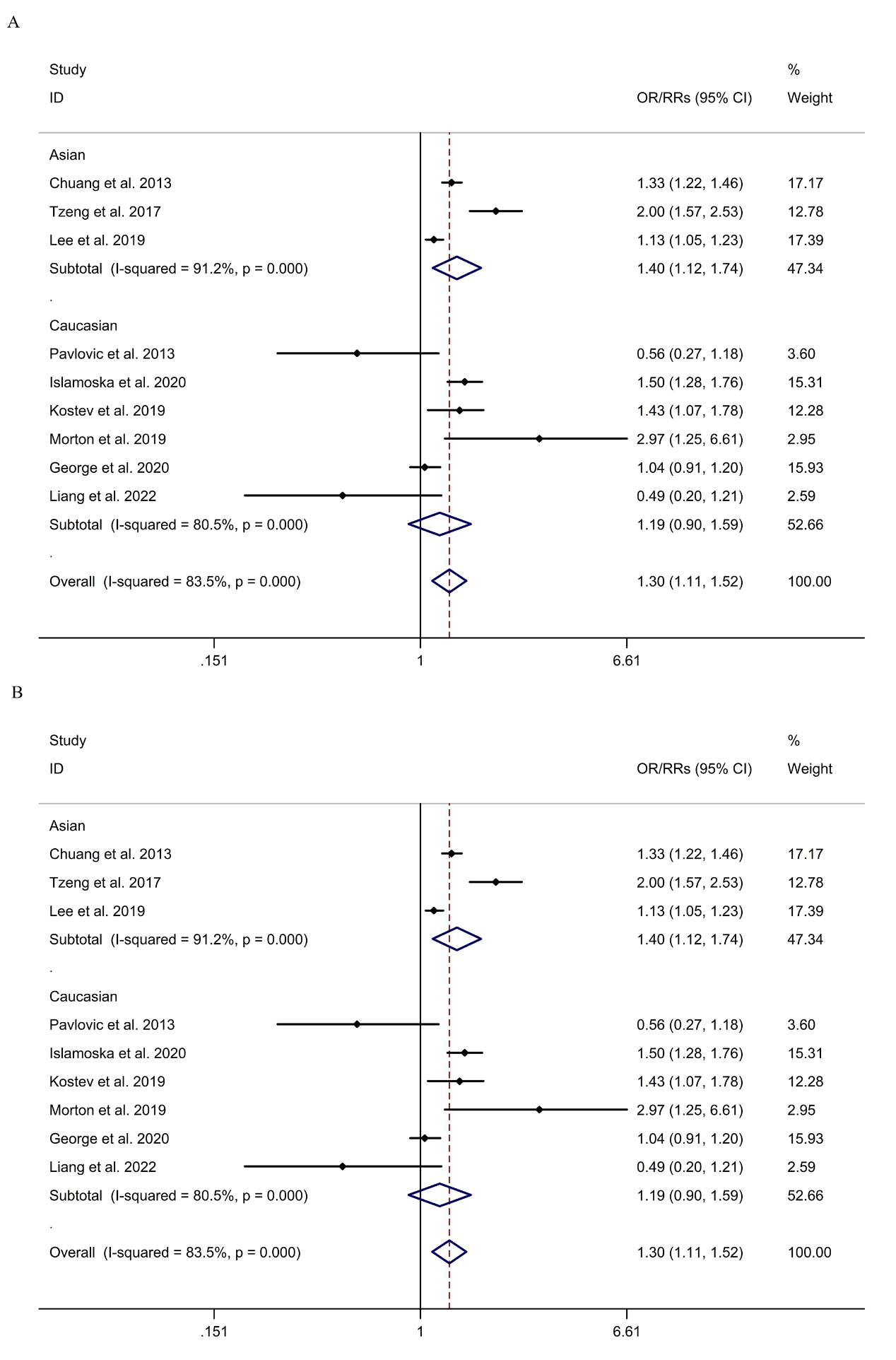


Supplementary figure 13. Subgroup analysis regarding association between migraine and risk of dementia in different ethnicities. Abbreviations: CI, confidence interval; OR, odds ratio; RR, relative risk.
