# Supplementary material for: Association between migraine and cognitive impairment
Source: J Headache Pain. 2022 Jul 26;23(1):88. doi: 10.1186/s10194-022-01462-4 (PMC9317452; doi:10.1186/s10194-022-01462-4)
Supplement: Supplementary file 18 — Additional file 18: Figure S14. Forest plots regarding association between MWoA and risk of dementia, migraine and risk of VaD, migraine and risk of AD. Abbreviations: AD, Alzheimer’s disease; CI, confidence interval; MWoA, migraine without aura; OR, odds ratio; RR, relative risk; VaD, vascular dementia. [file 10194_2022_1462_MOESM18_ESM.docx]

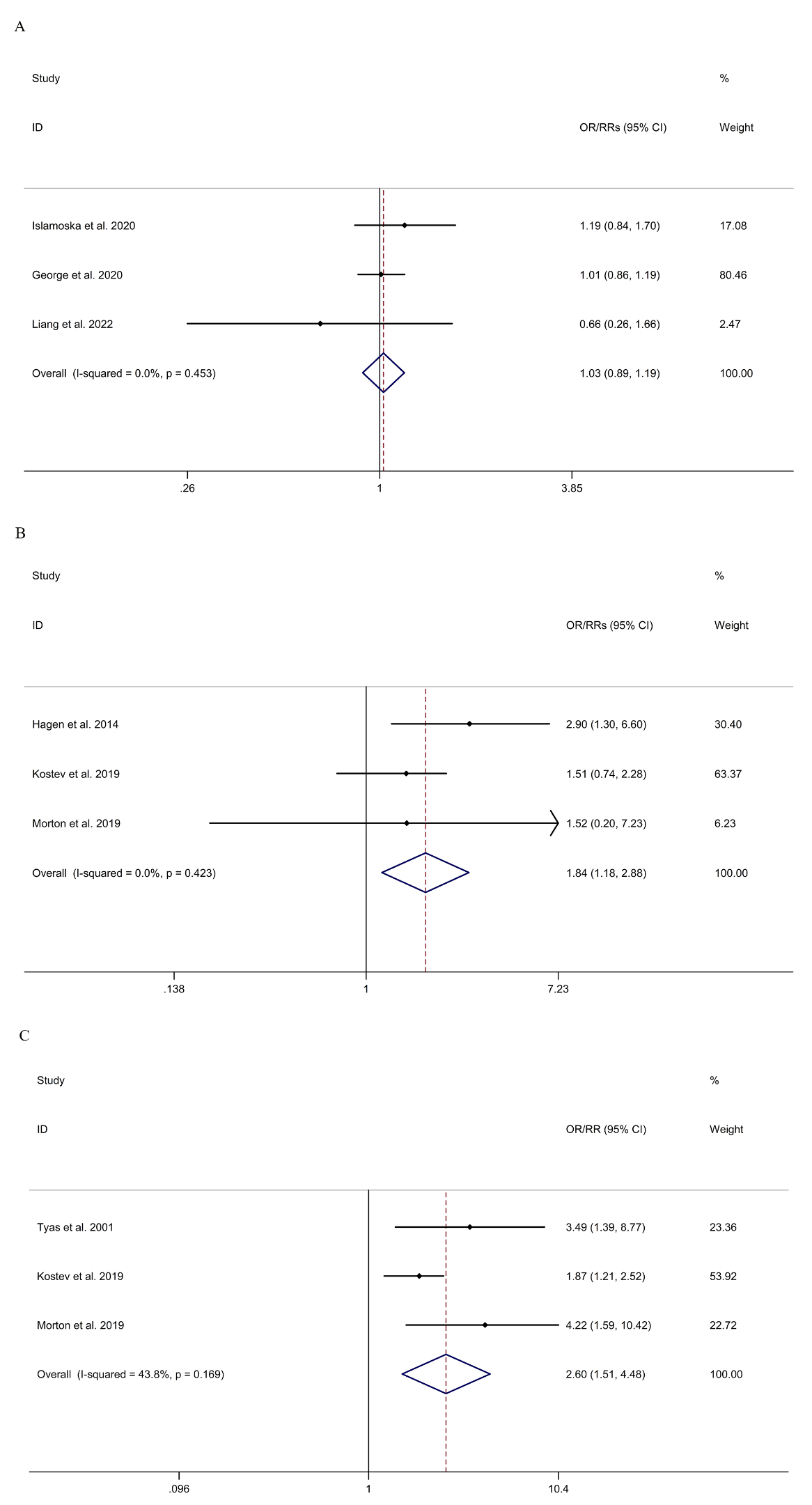


Supplementary figure 14. Forest plots regarding association between MWoA and risk of dementia, migraine and risk of VaD, migraine and risk of AD. Abbreviations: AD, Alzheimer’s disease; CI, confidence interval; MWoA, migraine without aura; OR, odds ratio; RR, relative risk; VaD, vascular dementia.
